# Benign masseter muscle hypertrophy

**DOI:** 10.1016/S1808-8694(15)31393-8

**Published:** 2015-10-17

**Authors:** Daniel Zeni Rispoli, Paulo M. Camargo, José L. Pires, Vinicius R. Fonseca, Karina K. Mandelli, Marcela A.C. Pereira

**Affiliations:** 1 Head of the ENT and Head and Face Surgery Department at Hospital Angelina Caron; 2 Head of the Larynx and Ear Ward of the ENT and Head and Face Surgery Department at HAC; 3 Head of the Otoneurology and Tinnitus Ward of the ENT and Head and Face Surgery Department at HAC; 4 Supervisor of the Pediatric ENT Ward at HAC; 5 Student at the ENT and Head and Face Surgery Department at HAC; 6 Student at the ENT and Head and Face Surgery Department at HAC. This paper was produced by the ENT and Head and Face Surgery Department at Hospital Angelina Caron

**Keywords:** anxiety, hypertrophy, muscle

## Abstract

Idiopathic hypertrophy of the masseter muscle is a rare disorder of unknown cause. Some authors associate it with the habit of chewing gum, temporo-mandibular joint disorder, congenital and functional hypertrophies, and emotional disorders (stress and nervousness). Most patients complain of the cosmetic change caused by facial asymmetry, also called square face, however, symptoms such as trismus, protrusion and bruxism may also occur. The goals of the present investigation were: to report a case of idiopathic masseter hypertrophy, describe its symptoms and treatment. The patient reported bilateral bulging in the region of the mandible angle, of slow and progressive evolution. He did not complain of pain or discomfort, however there was bilateral otalgia, nighttime trismus and stress. In his physical exam we noticed bilateral masseter hypertrophy without local inflammatory alterations. We indicated surgical treatment with an extraoral approach. Complementary tests are indicated when there is diagnostic doubts. Treatment varies from conservative to surgical, and the later depends on surgeon skill and experience.

## INTRODUCTION

Idiopathic masseter muscle hypertrophy (IMMH) was first described by Legg in 1880, reporting on the case of a 10-year-old girl with concurrent idiopathic temporalis muscle hypertrophy^1^, ^2^, ^3^, ^4^, ^7^. The masseter muscle is essential for adequate mastication and is located laterally to the mandibular ramus, and thus plays an important role in facial esthetics. A hypertrophied masseter will alter facial lines, generating discomfort and negative cosmetic impacts for many patients^1^, ^2^. Muscle function may also be impaired, thus introducing conditions such as trismus, protrusion, and bruxism^2^, ^3^. The etiology of this condition remains obscure^1^, ^2^, ^3^, ^4^, ^5^, ^6^, but some authors have correlated it to gum chewing, psychological disorders, and temporomandibular joint disorders, to name a few. It may affect anyone, regardless of age, gender and ethnicity^1^, ^2^, ^3^, ^6^, and also involve both sides of the face1–6. Differential diagnosis requires clinical history and physical examination and may even include complementary imaging resources such as CT scans to exclude other lesions^1^, ^5^. Differential diagnosis must include muscle tumors, salivary gland diseases, parotid tumors, parotid inflammatory diseases, and intrinsic masseter muscle myopathy^1^, ^2^, ^4^. Treatment can be conservative (using tranquilizers, mouthguards to minimize teeth clenching, psychological counseling etc^1^, ^3^, ^4^) or surgical (intraoral and extraoral approaches^1^, ^2^, ^3^, ^5^). This paper describes one case of idiopathic masseter muscle hypertrophy, reviews the literature and proposes an effective treatment option for the patient.

## LITERATURE REVIEW

### Incidence

IMMH is considered to be a rare disease, in spite of the growing esthetic concern manifested around it by patients^2^. People of Asian descent are more frequently involved^8^. According to a review of 108 cases described in the literature until 1984, Baek found that patient average age was 30 years, 57% were males, 60% had bilateral involvement, and only five patients had concurrent temporalis muscle hypertrophy^13^.

### Etiology

This origin of this condition is still unknown^1^, ^2^, ^3^, ^4^, ^5^, ^6^. Some authors have correlated idiopathic masseter muscle hypertrophy to a variety of conditions, such as defective teeth, dysfunctional mastication, gum chewing, TMJ disorders, teeth grinding, bruxism, and clenching teeth during sleep^2^. Therefore, anyone with the above mentioned conditions may develop unilateral or bilateral masseter muscle hypertrophy. People with psychological disorders or emotional disturbances that impact proprioception and the ability to keep the tone of the masseter muscle are at a higher risk of evolving to IMMH. According to Teixeira, there are two types of masseter muscle hypertrophy: congenital or familial and acquired due to functional hypertrophy^2^.

### Differential diagnosis

Idiopathic masseter muscle hypertrophy must be accurately diagnosed, as it may be mistaken for other diseases. Among them are unilateral compensatory hypertrophy (due to hypotrophy or hypoplasia in the contralateral side), masseter tumor, salivary gland disease, parotid tumor, parotid inflammatory disease, and masseter muscle intrinsic myopathy^1^, ^2^, ^4^.

### Diagnosis and symptoms

Diagnosis can be produced from clinical examination, directed interview, panoramic x-ray^1^, ^2^, ^3^, ^4^, ^5^, ^6^, and muscle palpation. This last diagnostic test consists of palpating the muscle with the fingers while the patient clenches his/her teeth so the muscle is more prominent during contraction. With the muscle relaxed and the patient’s mouth slightly open, extraoral palpation with both hands will pinpoint the intramuscular location of the hypertrophy. Under relaxation, the jaw angle may reveal irregularities that on the x-ray image may appear to be a bone increase^2^. According to Seltzer and Wang (1987), CT and MRI scans produce excellent images for the diagnosis of various masseter muscle conditions^9^. According to Maxwell e Weggoner (1951)^10^, neurologic tests and electromyograms are not useful in diagnosing this condition.

Most patients only complain of cosmetic problems, as an increased masseter introduces facial asymmetry, also called ‘square’ face^1^, ^2^, ^3^, ^4^, ^5^, ^6^. Some individuals complain of pain, headache, muscle stress, trismus, and intermittent masticatory claudication^1^, ^2^, ^3^, ^4^.

### Treatment

Clinical treatment is based on psychological counseling for patients with psychiatric disorders, use of mouthguards, antispasm and ansiolytic drugs, analgesics, and physical therapy^1^, ^3^, ^4^. Results are good for patients with mild hypertrophy. There is no reliable report on the literature on the success rates of isolated clinical therapy.

Surgical treatment was proposed for the first time by Gurney in 1947. The procedure consists of a sub-mandibular incision and the removal of 3/4 to 2/3 of all muscle tissue available from the muscle upper aponeurosis to the lower mandibular border^11^.

Mandibular angle osteotomy was supported by Adams in 1950^1^.

Removal of the masseter muscle insertion by means of a triangular incision was employed by Martensson in 1950 in a patient with history of bruxism and unilateral masseter muscle hypertrophy^12^.

Seventeen patients were surgically treated using the intraoral approach by Beckers in 1977. An internal muscle band was removed from the hypertrophied masseter (from the upper insertion in the zigomatic arc to the lower insertion in the mandibular angle), thus avoiding the production of a visible scar on the patient’s face and reducing the possibility of injuring branches of the facial nerve^14^.

The extraoral approach is often used to repair IMMH. The procedure is carried out through a sub-mandibular incision (Risdon) followed by the removal of an internal vertical band of the masseter muscle equivalent to approximately 2/3 of its thickness^1^.

## CASE REPORT

**Figure 1:**
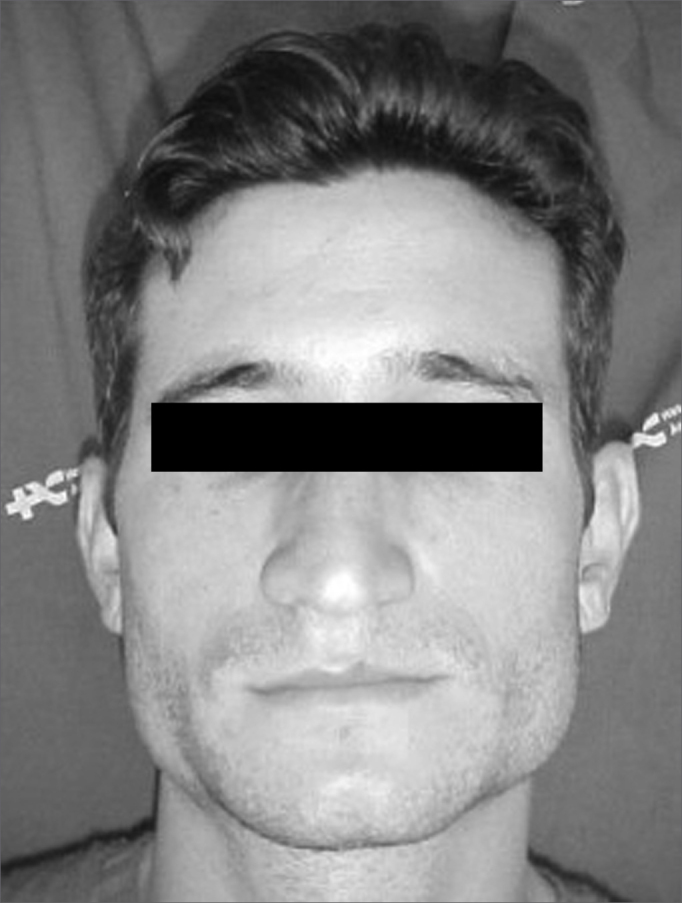
Patient with bilateral masseter muscle hypertrophy.

Patient M.A.S., aged 24, came to the Otorhinolaryngology Department complaining of bilateral increase of the mandibular angle region with onset six years prior to the visit (Fig. 1). The patient claimed the area was growing slowly but steadily, yet painlessly to palpation, and that it did not limit mouth opening. The patient also complained of bilateral otalgia, nocturnal trismus, and anxiety.

Physical examination revealed the patient had bilateral masseter muscle hypertrophy without further alteration. Data from clinical history and physical examination (intraoral and extraoral inspection and palpation led to the diagnosis of bilateral idiopathic masseter muscle hypertrophy.

The patient had no family history of masseter hypertrophy. Surgery was offered for cosmetic and functional reasons.

Procedure description:

1) A sub-mandibular incision (Risdon) of approximately 5 cm was made isolating the mandibular marginal nerve and the facial vessels;
2) The muscle was incised approximately 5 mm above the mandibular basilar. The entire ascending branch of the masseter was detached, and a vertical internal muscle band equivalent to 2/3 of the thickness of the muscle was resected;
3) After the muscle was resected, the remaining external third was sutured to its site of origin onto the muscle stump inserted in the mandibular basilar;
4) The bed was drained at the end of the procedure and a compressive dressing was put in place. The drain was removed 24 hours after the surgery;
5) Physical therapy was offered between the 10th and 14th day post-operatively.

## DISCUSSION

IMMH is a rare condition, but an increasing number of cases and surgical techniques have been recently described in the literature. The causes of IMMH require further clarification, but certain conditions seem to be associated with masseter hypertrophy such as psychological disorders, gum chewing, and dysfunctional TMJ. Many authors have reported that anxiety is often present in IMMH patients. The trismus experienced by our patient was related to stress and anxiety episodes. According to the literature, cosmetic alterations are the main complaint of IMMH patients. Our patient, however, complained of otalgia associated with TMJ disorder. Papers in the literature also describe masseter muscle and TMJ functional disorders as conditions associated with IMMH^1^, ^2^, ^3^, ^4^, ^5^, ^6^.

IMMH diagnosis is eminently clinical, and is based on identification of symptoms and cosmetic facial alterations connected to the progress of the disease. Physical examination through intra and extraoral palpation of an inflammation-free muscle serve to support the diagnosis. The main items to be considered in differential diagnosis are tumors in the large salivary glands (such as the parotid and submandibular), bone tumors in the middle and lower third of the face, muscle and salivary inflammatory processes, and nodular swelling. In cases of diagnostic doubt Seltzer and Wang (1987) recommend complementary exams such as CT and MRI scans that may help greatly in the diagnosis of masseter muscle conditions, salivary gland diseases, and bone or vascular tumor processes9. According to Maxwell and Weggoner (1951) neurologic tests and electromyography are not required.

IMMH surgical treatment is based on intra and extraoral approaches. Both techniques revolve around the removal of excessive muscle fibers from the inner third of the masseter vertical muscle fibers^1^, ^2^. Reduction osteoplasty may be performed in cases of bony hyperplasia of the mandibular angle. The remaining external bundle of the masseter should be attached to the mandibular periosteum to allow for adequate functional recovery. (1/2). The choice between intra and extraoral approaches, in our opinion, is not related to cosmetic or functional outcomes or to the risk of introducing vascular and nerve injury, but to the skill and experience of the surgeon in performing surgery using either of the approaches. Although Da Cruz et al. (1994) believe the intraoral approach offers lower risk of lesion and better cosmetic results, a surgeon with good experience in jaw procedures has no difficulties in isolating the mandibular nerve. The cosmetic outcome of submandibular incisions (e.g.: Risdon) is well accepted by the patients.

## CONCLUSION

IMMH is a disease of obscure etiology that may involve anyone. Although the diagnosis is eminently clinical, complementary exams may aid in the differential diagnosis against other conditions. The chosen surgical treatment will rely heavily on surgeon experience and skill.
